# Diffusion Mechanism of Aqueous Solutions and Swelling of Cellulosic Fibers in Silicone Non-Aqueous Dyeing System

**DOI:** 10.3390/polym11030411

**Published:** 2019-03-04

**Authors:** Liujun Pei, Yuni Luo, Xiaomin Gu, Huashu Dou, Jiping Wang

**Affiliations:** 1 Schhool of Fashion Engineering; Shanghai University of Engineering Science, Shanghai 201620, China; abbiexiaowang@hotmail.com; 2 Key Laboratory of Fluid Transmission Technology of Zhejiang Province; Zhejiang Sci-Tech University, Hangzhou 310018, China; huashudou@zstu.edu.cn; 3 Engineering Research Center for Eco-Dyeing and Finishing of Textiles; Zhejiang Sci-Tech University, Hangzhou 310018, China; 18108048319@163.com

**Keywords:** non-aqueous medium, cellulosic fiber, siloxane, diffusion, swelling

## Abstract

The main goal of this article is to study the diffusion mechanism of aqueous solutions and the swelling of cellulosic fibers in the silicone non-aqueous dyeing system via fluorescent labeling. Due to non-polar media only adsorbing on the surface of fiber, cellulosic fiber could not swell as a result of the non-polar media. However, because water molecules can diffuse into the non-crystalline region of the fiber, cellulosic fiber could swell by water which was dispersed or emulsified in a non-aqueous dyeing system. To study the diffusion mechanism of an aqueous solution in the siloxane non-aqueous dyeing system, siloxane non-aqueous media was first diffused to the cellulosic fiber because of its lower surface tension. The resulting aqueous solution took more time to diffuse the surface of the cellulosic fiber, because water molecules must penetrate the siloxane non-aqueous media film. Compared with the fluorescent intensity of the fiber surface, the siloxane film could be re-transferred to the dye bath under the emulsification of the surfactant and the mechanical force. Therefore, a longer diffusion time of the aqueous solution ensured the dyeing feasibility for cellulosic fiber in the non-aqueous dyeing system.

## 1. Introduction

Nowadays, non-aqueous dyeing systems are a promising new ecological dyeing technology which uses non-polar media to substitute for the traditional water base [1,2,3]. Non-aqueous dyeing media can neither dissolve the water soluble dye nor water, but it can transport material and transfer energy. The dye, alkali agent, and other dyeing auxiliaries are dissolved in a small amount of water which is dispersed or emulsified in non-polar media [4]. Only a small amount of aqueous solution is needed in the dyeing system because the dye and other chemical agents dissolve, and the swelling of fiber just needs a little water. Furthermore, the aqueous solution has a strong affinity to fiber and an aversion to non-polar media in the non-aqueous dyeing system [5]. As a result, the entire aqueous solution can be absorbed by the hydrophilic fibers. 

**Figure 1:**
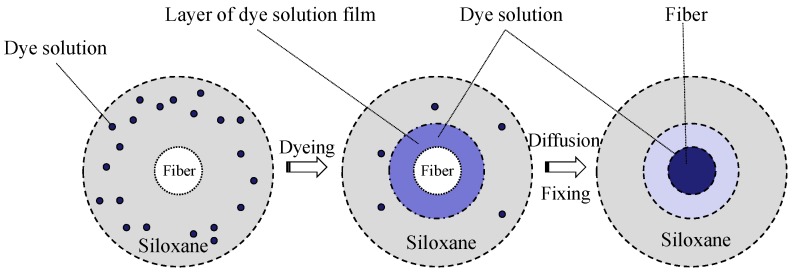
Schematic diagram of reactive dye in a siloxane non-aqueous dyeing system.

In our previous investigations [6,7,8,9,10], the siloxane non-aqueous medium (Decamethyl cyclopentasiloxane) was chosen as a continuous phase medium to prepare the siloxane non-aqueous dyeing system for dyeing natural fiber with reactive dye [11]. Siloxane non-aqueous medium is a clear, odorless, colorless, and non-oily cyclic siloxane fluid, which is widely used in consumer and industrial applications [12,13,14]. The boiling point of siloxane is about 210 °C which is higher than water. The surface tension of siloxane non-aqueous medium is very low (19.4 mN/m), resulting in it spreading on the fiber surface quickly. Moreover, research has demonstrated that siloxane non-aqueous medium is safe both to human health and the environment. In recent years, siloxane non-aqueous medium has been applied widely in dry cleaning [15]. We have used this system to dye cotton fiber with reactive dye. As shown in Figure 1, the dye particles are evenly suspended in the siloxane medium with the aid of the dispersants. Since the reactive dye solution is completely non-miscible with siloxane but has a strong affinity to water, reactive dye particles will quickly diffuse to the thin layer of free water (alkali solution) on fiber surface. As a result, almost all of the dye will be transferred to the fiber’s surface and dissolved in the water film under mechanical force. The final uptake of reactive dye in siloxane media is close to 100%, which is relatively higher than traditional water system (60–70%) [11]. Furthermore, the fixation rate for this process is also higher than that of traditional process (80–90% for siloxane media vs. 40–60% traditionally) [6,10]. Compared with the traditional water based dyeing, the mechanical and thermal properties of cellulosic fibers changed a little. After dyeing, the siloxane non-aqueous media can be recycled and reused by a simple static separation.

However, the affinity between dye and fiber might influence the level dyeing property, defined as a characteristic of dye distribution uniformly on the substrate in a dyeing system [12,13]. In particular, whether the dye or non-aqueous media film which adsorbed on the fiber’s surface can be re-migrated into a dye bath, the influence of siloxane film on the diffusion of dye, and the swelling of fiber have not been systematically studied.

The main object of this research is to study the diffusion mechanism of water soluble dye in the siloxane medium using two fluorescent dyes (Coumarin 6 and Rhodamine B). The diffusion of water soluble dye, the migration of dye and non-aqueous media film from fiber to dye bath, and the distribution of siloxane/aqueous solution in the interior of the fiber were investigated with Confocal Microscope analysis. In addition, fiber swelling in different non-aqueous media and traditional water base were also systematically studied.

## 2. Experimental

### 2.1. Materials

100% cotton yarn (32s) and viscose yarn (32s) were obtained from Huafu Top Dyed Melange Yarn Co., Ltd. (Shangyu, China). Rhodamine B and Coumarin 6 were purchased from Aladdin Reagent Co., Ltd. (Shanghai, China). Alkyl alcohol polyoxyethylene ether (AEO-3, CP) was obtained from Tianjing Haoyuan Chemical Co., Ltd. (Tianjin, China). Siloxane non-aqueous medium (purity ≥ 98%) was purchased from GE Toshiba Silicone Ltd. (Jiande, China). Paraffin, C_8_H_18_ (isooctane) and Ethanol (C_2_H_5_OH), all with purity above 99.7%, were purchased from Hangzhou Gaojing Chemical Reagent Co., Ltd. (Hangzhou, China). The parameters of different dyeing media are shown in Table 1.

### 2.2. Preparation of Fluorescent Solution

To prepare 1 g/L fluorescent solution, 0.10 g Coumarin 6 was dissolved in 100 mL each of siloxane, paraffin, isooctane, and ethanol media. Furthermore, 0.10 g Rhodamine B was dissolved in 100 mL distilled water to mark the aqueous solution.

### 2.3. Fiber Swelling Evaluation

Swelling was performed with an approximate fiber to liquid weight ratio of 1:50. All fiber samples were treated with different media at 25 °C for 1 min and 60 °C for 20 min in a beaker. A confocal microscope (Nikon C2, Tokyo, Japan) was used to measure the diameter change of fiber during the swelling process. The instrument included two panels: The bright field channel, and the laser channel. The laser channel contained four excitation wavelengths: 405, 488, 543 and 640 nm. The changing of the fiber’s diameter was measured under the bright field channel and two laser channels (488 nm and 543 nm).

### 2.4. Disffusion of Reactive Dye Solution in the Siloxane Non-Aqueous Dyeing System

To observe the diffusion of the reactive dye solution in the siloxane non-aqueous dyeing system, the distribution of the aqueous solution in the cellulosic fiber was measured with the confocal microscope at 200–400x.

**Figure 2:**
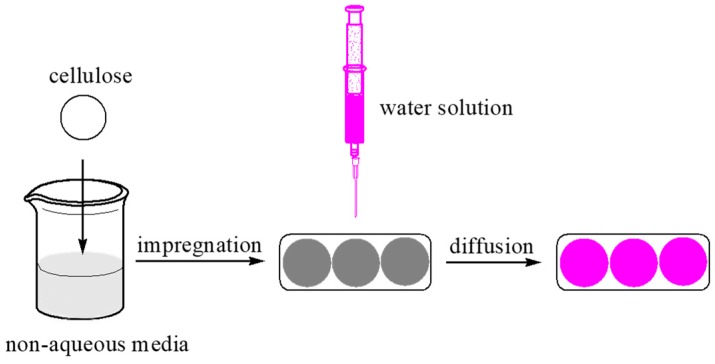
A schematic diagram of reactive dye solution diffusion in the siloxane non-aqueous dyeing system.

Cellulosic fiber was first impregnated in non-aqueous media for 1 min. Then the diffusion of water solution (0.2 µL for each time) on cellulosic fiber surface was measured with a Video Contact Angle Tension Meter (Kruss GmbH DAS20, Hamburg, Germany). The beginning and the end of the diffusion time were recorded simultaneously. The schematic diagram is shown in Figure 2.

Wetting time of non-aqueous media and water solution on cellulosic film was recorded using the DAS20 as well. The time difference was recorded between the beginning of the wetting and the end of the wetting during the whole wetting process.

### 2.5. The Migration of Siloxane Media on Viscose

Small viscose yarn (0.10 g) was soaked in 5 mL siloxane (1 g/L Coumarin 6) solution at 25 °C for 20 min and dried at 60 °C for 48 h. After drying, washing simulation was done by the flowing of the aqueous solution or surfactant solution (AEO-3, 0.10 g/L) with a flow rate of 200 μL/min. Confocal microscopy was used to measure the change of fluorescence intensity on the fiber’s surface at different washing times (1, 3 and 5 min).

## 3. Results and Discussion

### 3.1. Fiber Swelling

**Figure 3:**
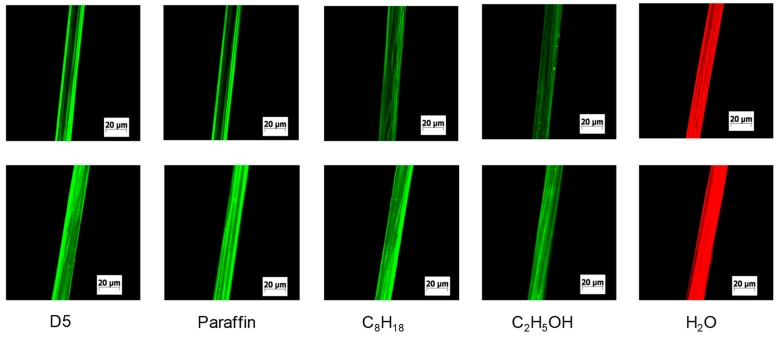
Confocal fluorescence images of viscose in different media: The fibers which were treated at 25 °C for 1 min as the control samples (top row); and the fibers which were treated at 60 °C for 20 min as the swelled samples (bottom row).

Fiber swelling is an important factor for the reactive dyeing. A good fiber swelling can improve dye penetration; otherwise, reactive dye just adsorbs on the surface of the fiber and cannot penetrate into the interior of the fiber. Furthermore, an increase in the swelling of cellulosic fibers improves their flexibility and dyeing properties [14,15,16]. The method for determining the swelling of cellulosic fiber is the measurement of its centrifugal water retention value [17,18]. This method is frequently used because of simplicity, but it cannot directly show the diameter change of the fiber. As shown in Figure 3, according to the fluorescence intensity of fiber surface, Rhodamine B (water media) and Coumarin 6 (siloxane media) diffused to the surface of the fiber. Therefore, the change in fiber diameter can be measured with the fluorescent labeling.

Because fiber is difficult to swell at low temperature [16], the diameter of fiber was defined using a control sample which was treated with non-aqueous media or water at 25 °C for 1 min (top row of Figure 3). Figure 3 shows the change of viscose diameter in different media. The degree of fiber swelling was calculated using Equation (1).

**Figure 4:**
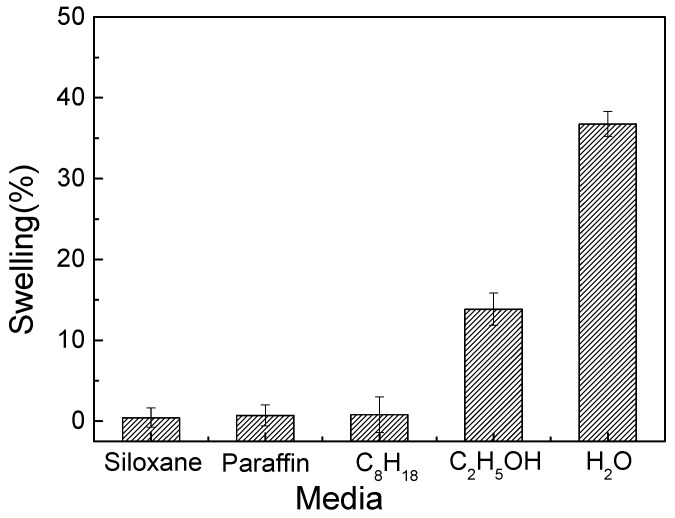
The swelling of viscose in different media.

S_w_ (%) = (d_t_−d_0_)/d_0_ × 100% (1)

where Sw (%) refers to the fiber swelling in different media, d_t_ (μm) refers to the fiber diameter at 60 °C for 20 min, d_0_ (μm) refers to the initial fiber diameter at 25 °C for 1 min. The results are shown in Figure 4.

As shown in Figure 4, the change of viscose diameter was relatively low in the siloxane, paraffin, and C_8_H_18_ media, indicating that fiber could not swell in these non-aqueous media. However, the swelling degree of viscose was 13.8% and 36.8% in the ethanol and water respectively. These results showed that viscose could swell sufficiently in the ethanol and water bath.

**Figure 5:**
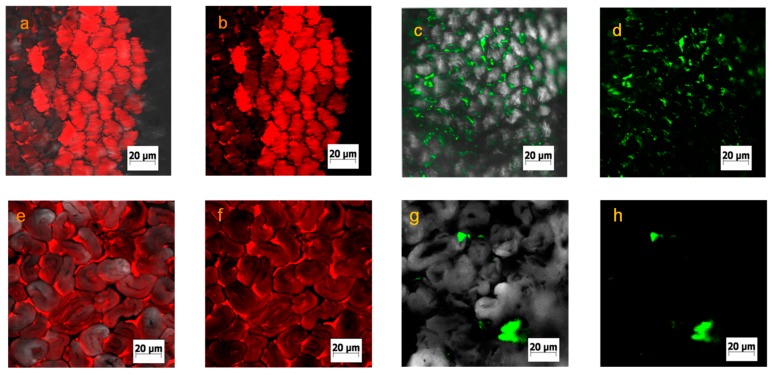
Confocal fluorescence images of distribution of water (**a**, **b**,**e**,**f**) and siloxane (**c**,**d**,**g**,**h**) medium in viscose (**a**,**b**,**c**,**d**) and cotton (**e**,**f**,**g**,**h**) fiber; the merged channel (**a**,**c**,**e**,**g**) and the laser channel (**b**,**d**,**f**,**h**).

Many parameters influence the swelling of fiber, including fiber type, temperature, time, etc. [19,20]. In non-aqueous media, the chemical composition of the solution would influence the fiber swelling, as well as the polarity of the media, and the molecular size [21,22]. Polar groups are not contained in the molecular structures of siloxane, paraffin, and isooctane. However, for ethanol and water, their molecules contain some polar groups (–OH) which can form hydrogen bonds with cellulosic fiber. Moreover, it was clearly noticed that Rhodamine B diffused to the interior of viscose and cotton (Figure 5a,b,e,f), indicating that water molecule could diffuse to the interior of the fiber. For siloxane dyeing media, Coumarin 6 only diffused to the surface or interval fiber (Figure 5c,d,g,h), indicating that the siloxane molecule could not permeate to the interior of the fiber. This may be the reason why water can swell fiber, but the siloxane non-aqueous medium cannot. 

### 3.2. The Wetting Time of Non-Aqueous Media and Aqueous Solution on Cellulosic Film

For the wetting time of different non-aqueous media and aqueous solutions on cellulosic film (Table 2), other non-aqueous media (siloxane, paraffin and isooctane) can quickly diffuse on the cellulosic film in addition to ethanol. However, the aqueous solution and ethanol took 2–3 seconds to diffuse on the cellulosic film. From Table 1, it can be known that the surface tension of non-aqueous media is in the order of siloxane < paraffin < C_8_H_18_, which is related to their diffusion time on cellulosic film. A lower surface tension may result in a shorter wetting time, however, viscosity may also affect the diffusion time of non-aqueous media [23,24]. Therefore, the diffusion of paraffin is greater than that of isooctane.

### 3.3. Influence of Non-Aqueous Media on the Adsorption of Aqueous Solution

**Figure 6:**
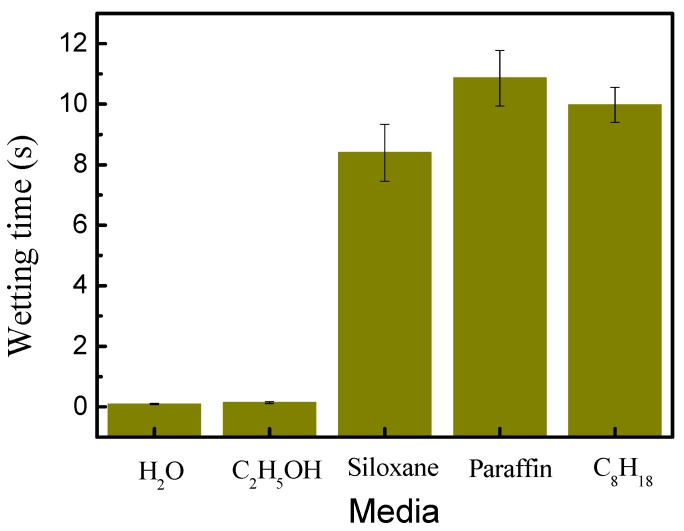
Influence of non-aqueous media on the adsorption of the aqueous solution.

The wetting time of the non-aqueous media and the aqueous solution indicated that the non-aqueous medium is the first to diffuse to the cellulosic fiber. Figure 6 shows the effect of non-aqueous media on the adsorption of the aqueous solution on cellulosic film. After impregnating with water or ethanol, the aqueous solution can be quickly diffused on the cellulosic film surface. However, if the cellulosic film was impregnated with siloxane, paraffin or isooctane, the diffusion of the aqueous solution on cellulosic film surface needs more time (8–10 s).

According to the similar compatibility [25], if the cellulosic fiber was impregnated with a hydrophilic substance (water or ethanol), a reactive dye solution could quickly spread on the surface of cellulosic fiber. On the other hand, the surface of cellulosic fiber would change to lipophilic after treatment with siloxane, paraffin and isooctane. As a result, aqueous solutions have difficulty penetrating the lipophilic films due to their poor solubility in non-aqueous media, but due to gravity, aqueous solution eventually penetrate non-aqueous film and diffuse to a cellulosic fiber surface [26].

The Video Contact Angle Tension Meter was designed to observe the diffusion of a reactive dye solution on cellulosic fiber in the siloxane non-aqueous dyeing system. Cellulosic fiber was first dipped in non-aqueous media for 1 min, then 0.2 µL aqueous solution was injected to study the whole diffusion. 

**Figure 7:**
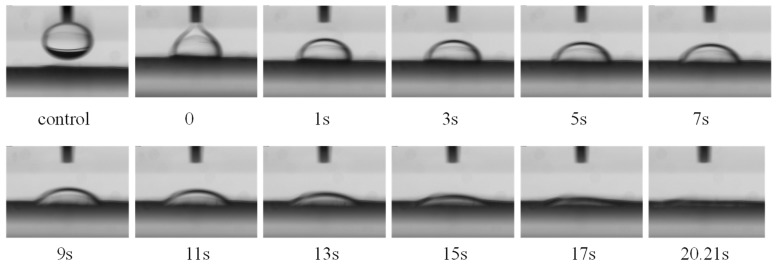
The diffusion of the water solution on the cellulosic fiber surface in the siloxane non-aqueous media.

As shown in Figure 7, the aqueous solution needed 20.21 seconds to diffuse on the cellulosic fiber surface in the siloxane non-aqueous media. Compared with the above results, due to the influence of the non-aqueous media film which adsorbed on the fiber’s surface, the aqueous solution took more time to complete the diffusion in the siloxane non-aqueous dyeing media. It is well known that oil and water are incompatible, but the density of water is more than the siloxane; as a result, water molecules can permeate the siloxane film and adsorb on the fiber surface [27,28,29]. Therefore, a longer diffusion time of the aqueous solution ensures the feasibility for dyeing cellulosic fiber in this process, and the level dyeing property is good in the siloxane non-aqueous dyeing system.

### 3.4. Migration of Non-Aqueous Media Film

**Figure 8:**
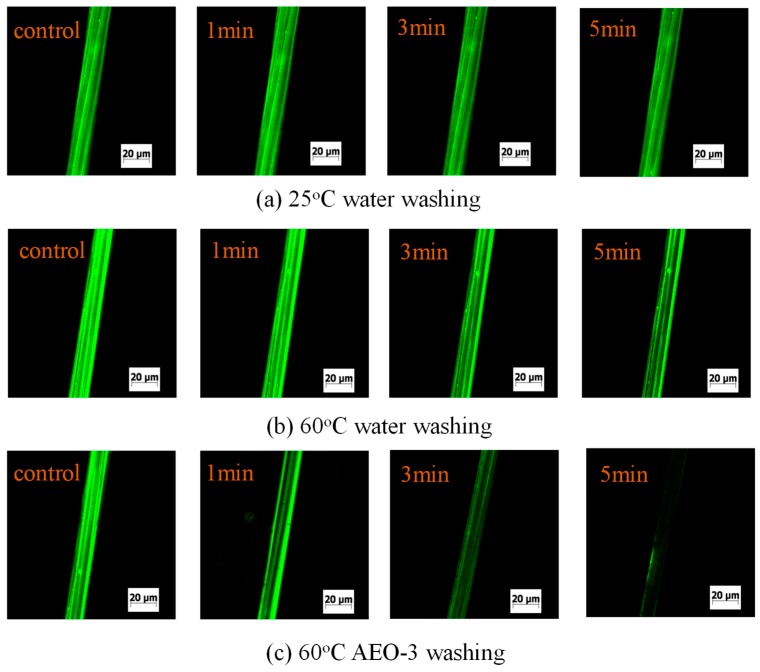
The change of FITC fluorescence intensity on the fiber surface under different washing conditions: (**a**) 25 °C water; (**b**) 60 °C water; (**c**) 60 °C AEO-3 aqueous solution.

To analyze the reason why cellulosic fiber can get a good level dyeing property in the siloxane non-aqueous dyeing system, the migration of siloxane film on the fiber surface was studied. Figure 8 shows the changing of fluorescein isothiocyanate (FITC) fluorescence intensity under different conditions. Compared with the control sample, FITC fluorescence intensity changed a little after washing of 25 °C or 60 °C water for 5 min, indicating that the interfacial siloxane film was difficult to migrate into the dye bath. However, the fluorescence intensity of Coumarin 6 changed significantly after washing 5 min with an AEO-3 solution, indicating that surface siloxane film can migrate into the dye bath with the aid of surfactant. 

According to our previous studies [7], if some surfactants were added in the siloxane non-aqueous dyeing system, the dyeing level property of the reactive dye was improved. From the theory of emulsification [30,31,32], the surfactant can reduce the surface tension of water. Hence, the siloxane film which adsorbs on the surface of fiber can migrate into the dyeing bath [33,34,35]. Moreover, reactive dyes can be redistributed by cellulosic fibers. As a result, a good level dyeing property could be obtained in the siloxane non-aqueous dyeing system.

## 4. Conclusions

This study investigates the diffusion mechanism of an aqueous solution and the swelling of cellulosic fiber in a non-aqueous dyeing system. In a non-aqueous dyeing system, the cellulosic fiber hardly swells due to the non-aqueous medium, but an aqueous solution which is dispersed or emulsified in a non-aqueous medium can sufficiently swell the cellulosic fiber. The main reason is that the non-polar medium has difficulty diffusing the interior of the fiber in the dyeing system. For the diffusion mechanism of an aqueous solution in the siloxane non-aqueous dyeing system, the siloxane non-aqueous medium can quickly diffuse in the cellulosic film, while an aqueous solution takes more time to diffuse the cellulosic film. Furthermore, the lower surface tension of the non-aqueous medium ensures the dyeing medium quickly diffuses on the fiber’s surface; as a result, the aqueous solution takes more time to penetrate the lipophilic film and diffuse on the cellulosic fiber surface. Most remarkably, comparing fluorescent intensity, the siloxane film is hardly washed off by the aqueous solution, and can be re-migrated to the dye bath under mechanical force and the emulsification of the surfactant.

## Figures and Tables

**Table 1:** The parameters of different dyeing media.

Medium	Surface Tension (dyn/cm)	Boiling Point (°C)	Viscosity (mm^2^/s)
Siloxane	18	210	5
Paraffin	19	330	16
C_8_H_18_	23	126	0.7
C_2_H_5_OH	22	78	1.1
H_2_O	72	100	1.0

**Table 2:** The wetting time of different dyeing media on cellulosic film.

Medium	Siloxane	Paraffin	C_8_H_18_	C_2_H_5_OH	H_2_O
Wetting time (s)	0.11	1.28	0.99	2.34	3.01
